# Global observation gaps of peatland greenhouse gas balances: needs and obstacles

**DOI:** 10.1007/s10533-023-01091-2

**Published:** 2023-10-15

**Authors:** Junbin Zhao, Simon Weldon, Alexandra Barthelmes, Erin Swails, Kristell Hergoualc’h, Ülo Mander, Chunjing Qiu, John Connolly, Whendee L. Silver, David I. Campbell

**Affiliations:** 1 Department of Biogeochemistry and Soil Quality, Division of Environment and Natural Resources, Norwegian Institute of Bioeconomy Research, Ås, Norway; 2 University of Greifswald, Greifswald, Germany; 3 Center for International Forestry Research (CIFOR), Bogor, Indonesia; 4 Center for International Forestry Research (CIFOR), Lima, Peru; 5 Centre de coopération International en Recherche Agronomique pour le Développement (CIRAD), UMR Eco&Sols, Montpellier, France; 6 Department of Geography, University of Tartu, Tartu, Estonia; 7 Research Center for Global Change and Complex Ecosystems, East China Normal University, Shanghai, China; 8 Laboratoire des Sciences du Climat et de l’Environnement, CEA-CNRS-UVSQ, Gif-sur-Yvette, France; 9 Department of Geography, School of Natural Science, Trinity College Dublin, Dublin, Ireland; 10 Department of Environmental Science, Policy & Management, University of California, Berkeley, CA USA; 11 School of Science, University of Waikato, Hamilton, New Zealand

**Keywords:** CO_2_, CH_4_, N_2_O, Eddy covariance, Chamber, Land use

## Abstract

Greenhouse gas (GHGs) emissions from peatlands contribute significantly to ongoing climate change because of human land use. To develop reliable and comprehensive estimates and predictions of GHG emissions from peatlands, it is necessary to have GHG observations, including carbon dioxide (CO_2_), methane (CH_4_) and nitrous oxide (N_2_O), that cover different peatland types globally. We synthesize published peatland studies with field GHG flux measurements to identify gaps in observations and suggest directions for future research. Although GHG flux measurements have been conducted at numerous sites globally, substantial gaps remain in current observations, encompassing various peatland types, regions and GHGs. Generally, there is a pressing need for additional GHG observations in Africa, Latin America and the Caribbean regions. Despite widespread measurements of CO_2_ and CH_4_, studies quantifying N_2_O emissions from peatlands are scarce, particularly in natural ecosystems. To expand the global coverage of peatland data, it is crucial to conduct more eddy covariance observations for long-term monitoring. Automated chambers are preferable for plot-scale observations to produce high temporal resolution data; however, traditional field campaigns with manual chamber measurements remain necessary, particularly in remote areas. To ensure that the data can be further used for modeling purposes, we suggest that chamber campaigns should be conducted at least monthly for a minimum duration of one year with no fewer than three replicates and measure key environmental variables. In addition, further studies are needed in restored peatlands, focusing on identifying the most effective restoration approaches for different ecosystem types, conditions, climates, and land use histories.

## Introduction

Peatlands store more than a third of global soil carbon but are predicted to shift from a sink to a source of carbon because of global warming and anthropogenic disturbance (Loisel et al. 2021). Since the 17th Century, peatlands have been extensively drained for agriculture, forestry, mining and urban development (Minasny et al. 2019), particularly in Europe and North America and more recently in Northeast and Southeast Asia. This land use conversion has resulted in significant peat carbon losses as CO_2_) and/or CH_4_ (e.g., Couwenberg et al. 2010; Frolking et al. 2011; Furukawa et al. 2005; Hatala et al. 2012). Many studies have also reported significant emissions of N_2_O, a potent GHG, being released from managed peatlands (e.g., Anthony and Silver 2021; Anthony et al. 2023; Oestmann et al. 2022; Ojanen et al. 2010; Parn et al. 2018) and permafrost soils (Voigt et al. 2020). There is increasing recognition of the importance of peatlands for their role in carbon storage, climate mitigation and water regulation (Andersen et al. 2017; Chimner et al. 2017; Dohong et al. 2018). For appropriate management of peatlands in different regions, it is important to assess GHG emissions under different land uses, including restoration of natural peatland functions.

Accurate estimates and predictions of peatland GHG emissions using models require high-quality observation data that capture temporal variations, such as diurnal and inter-annual changes, while providing comprehensive coverage of spatial variability on a global scale (Mozafari et al. 2023). Although there has been a notable increase in GHG flux observations in peatlands during the last decade, many regions with peatlands are still understudied. Hence, summarizing existing studies and identifying observation gaps are crucial steps in guiding future research.

Among methods for observing GHG fluxes, the closed (non-steady-state) chamber and eddy covariance (EC) technique are the most widely used approaches in ecosystem research (Schrier-Uijl et al. 2010; Shi et al. 2022). Chamber observations measure fluxes at the plot scale and are usually carried out in field campaigns. With a small footprint, chambers are suitable for studying processes underlying GHG fluxes through manipulative experiments and inspecting micro-spatial variability (e.g., hummock-hollow microtopography, fertilized versus non-fertilized areas). Because of a high mobility and relatively low cost, chamber measurements can be employed in remote locations with a modest investment (e.g., Glagolev et al. 2011; Reeburgh et al. 1998; Veber et al. 2018). By contrast, EC produces continuous sub-hourly flux data at the ecosystem scale, providing an excellent method for long-term GHG monitoring and annual budget estimation (Baldocchi 2020). However, EC instrumentation is usually very costly and requires stable power supply, and skilled labor for installation, maintenance, and data processing, which restricts its widespread application, particularly in remote areas. Overall, EC and chamber observations complement each other and ideally, should be used together to achieve more comprehensive GHG monitoring.

GHG flux measurements have been recorded at numerous peatland sites worldwide; however, these observations may not be representative of all peatlands globally, and observation gaps still exist in many regions due to factors such as limitations in site accessibility, instrument availability, skilled labor, research funding and regional/national policies. In this study, we carried out a literature search to collect studies that measured peatland GHG fluxes. The aims are to (1) summarize observation gaps in terms of the region, gas type, method, and land use type, (2) identify the underlying obstacles to widen the coverage of GHG observations, and (3) suggest future research directions.

## Materials and methods

### Data collection

Firstly, we carried out a literature search for publications during 2000–2022 using the Boolean search term “peatland AND (CO_2_ OR CH_4_ OR N_2_O)”. To ensure the quality of publications, the literature search was confined to the mainstream publishers that publish only peer-reviewed studies, including Elsevier, Wiley, Springer, PLOS, Oxford, Nature, PNAS, MDPI, IOP, Frontiers, Copernicus Publications, Science and Taylor & Francis. For publishers with a functional search engine that supports Boolean terms, the search was performed directly on the website of the publishers (i.e., Elsevier, Wiley, Springer, and Copernicus Publications). For other publishers, the search was conducted on Google Scholar using “Advanced search” and specified the publisher names. The search resulted in a total of 6,457 studies, from which we downloaded the title, publication year, abstract and the link. To include only papers related to field GHG flux observations, we further removed reviews/syntheses/meta-analysis, modeling or remote sensing studies and ex-situ observations (e.g., incubations, mesocosms, etc.). Papers that did not report the existence of organic soil layers at the study sites were also excluded. After filtering, we obtained a dataset of 914 studies, which represent the majority of existing publications on GHG flux observations in peatlands during 2000–2022 from mainstream peer-reviewed sources. It is noted that publications in other languages than English are not included.

### Data extraction

We extracted the following information for each study from the title and abstract: (1) country of the study site, (2) method(s) used for flux measurements, (3) land use type(s) (i.e., natural, drained, cultivated, abandoned, restored), and (4) whether permafrost or forest was present. If multiple land use types were investigated within a single study, each of them was treated as a distinct entry in the dataset. For studies not documenting this information in the title or abstract, the full text was downloaded for the information extraction.

Compared to the EC approach which has relatively strict instrumentation requirements, sampling design for chamber measurements vary greatly among studies (e.g., gas analyzers, chamber design, frequency, replicates, auxiliary measurements), resulting in large variabilities in the data quality. To evaluate the current convention and quality of chamber flux measurement, we further extracted the following information from the full text of studies published in 2022: (1) seasonal coverage, (2) measurement duration, frequency and replication, (3) equipment (chamber type and analyzer), (4) calculation methods and (5) whether environmental variables had been measured (i.e., radiation, air temperature, soil temperature, soil moisture, water table, soil pH, bulk density and soil carbon/nitrogen content).

### Data analysis and visualization

To identify national/regional research gaps, we compare the number of studies per country relative to its peatland area. The data of peatland distribution for each country were derived from the Global Peatland Database (Greifswald Mire Center). We computed a gap index for GHG observations per country as follows:

1 $${\text{Gap index = 1 - }}\frac{{{\text{Study density}}}}{{{\text{Max }}\left\{ {{\text{Study density}}_{{\text{1}}} {\text{,Study density}}_{{\text{2}}} , \ldots ,{\text{Study density}}_{{\text{j}}} } \right\}}}$$

where *j* is the number of countries with peatland distributions, and *Study density* for each country was calculated as:

2 $${\text{Study density = }}\frac{{{\text{n + 0}}{\text{.1}}}}{{{\text{ln}}\,\left( {{\text{Area}}} \right)}}$$

where *n* is the number of studies per country, *Area* is the national peatland area (kha), and *(n + 0.1)* is a correction to account for countries with no studies. The gap index ranges from 0 (no/small gap) to 1 (large gap) and a value above 0.9 was taken to indicate a significant need for GHG observations. We used the “ggplot2” package (Wickham 2016) in the program R 4.2.2 (R Development Core Team 2022) for data visualization.

## Results

### Flux gas types and methods

**Fig. 1:**
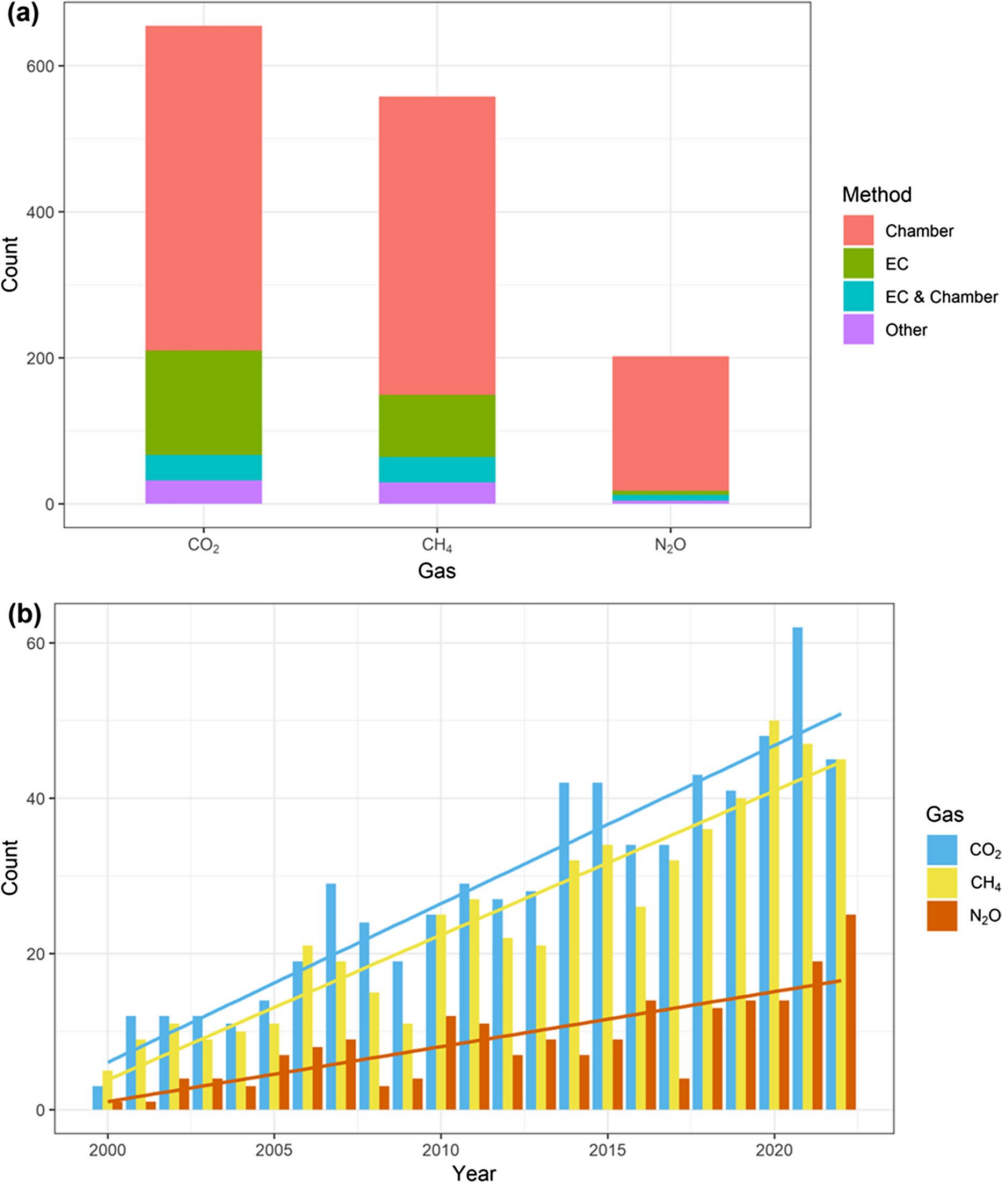
Number of studies that conducted CO_2_, CH_4_ and N_2_O flux measurements from peatlands (**a**) and their yearly distribution between 2000 and 2022 (**b**). Colors indicate the methods used for the measurements in (**a**) and different gas types in (**b**). Lines in (**b**) indicate the linear trends

Among the 914 peatland studies we collected, 655 studies measured CO_2_ flux, 558 studies measured CH_4_ flux, and 202 studies measured N_2_O flux (Fig. 1a). It is noteworthy that 355 studies (39%) measured both CO_2_ and CH_4_ fluxes and only 111 studies (12%) measured fluxes of all three gases. Most studies that measured CO_2_ flux used the chamber approach (480 studies or 73%), the remaining (178 or 27%) used the EC approach and 35 studies (5%) included observations using both chambers and EC. Only 32 studies (5%) used other methods, such as approaches based on Fick’s Law of diffusion (e.g., Denfeld et al. 2020). The CH_4_ flux studies exhibited similar patterns in their methods with 444 (80%) using the chamber approach, 120 (22%) using EC and 29 (5%) using other methods. For N_2_O, the vast majority of studies (192 or 95%) employed the chamber method. Only 14 (7%) and four studies (2%) used EC and other methods, respectively.

In 2000, the number of studies that measured either CO_2_, CH_4_ or N_2_O fluxes from peatlands was less than six (Fig. 1b). Over the period 2000–2022, the numbers of CO_2_ and CH_4_ flux studies increased at a rate of ~ 2.0 studies per year while that of N_2_O flux studies increased only by 0.7 studies per year.

### Country distribution

**Fig. 2:**
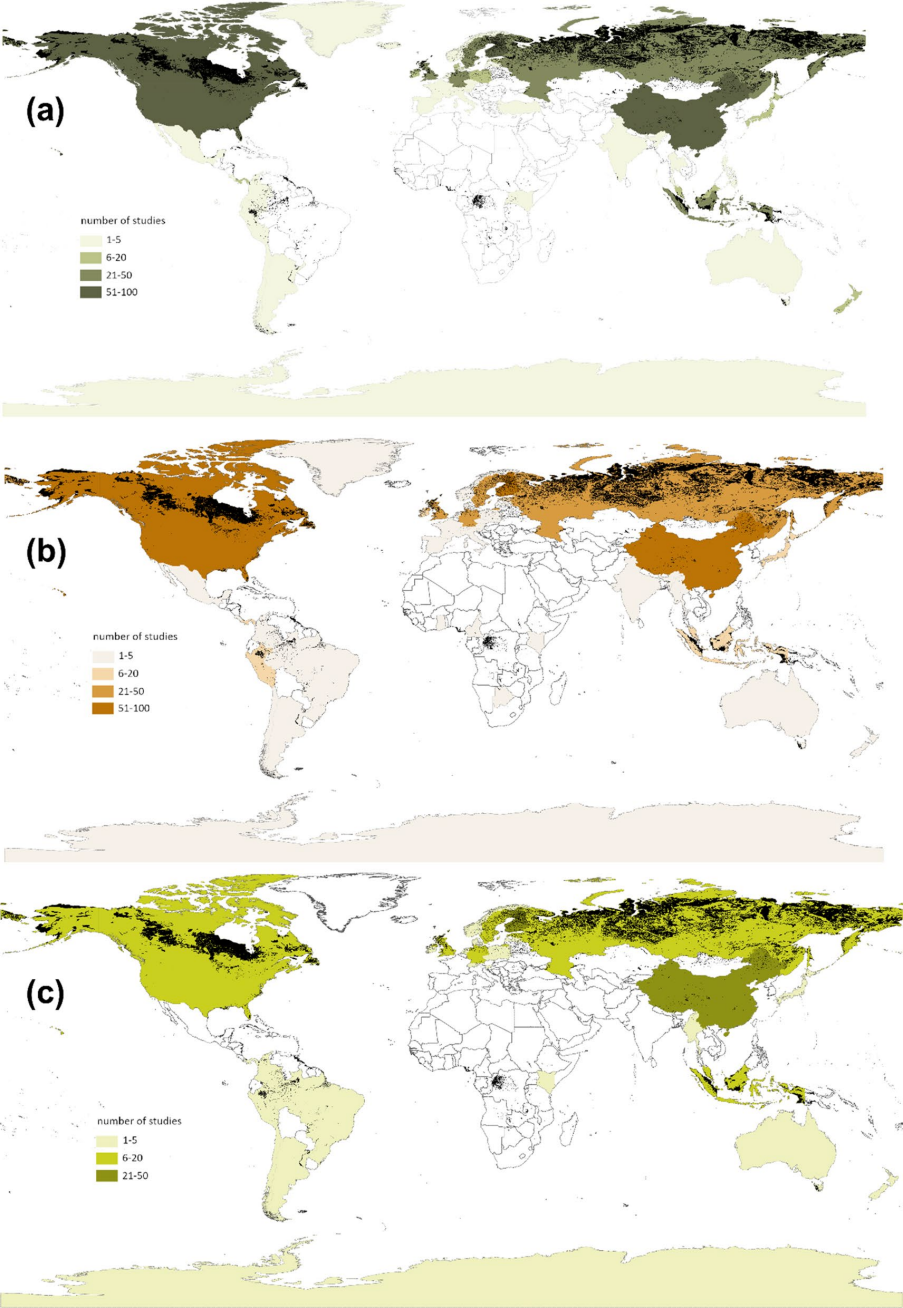
Number of studies that measured CO_2_ (**a**), CH_4_ (**b**) and N_2_O fluxes (**c**) in different countries. Areas with peatland distribution are marked in black

**Fig. 3:**
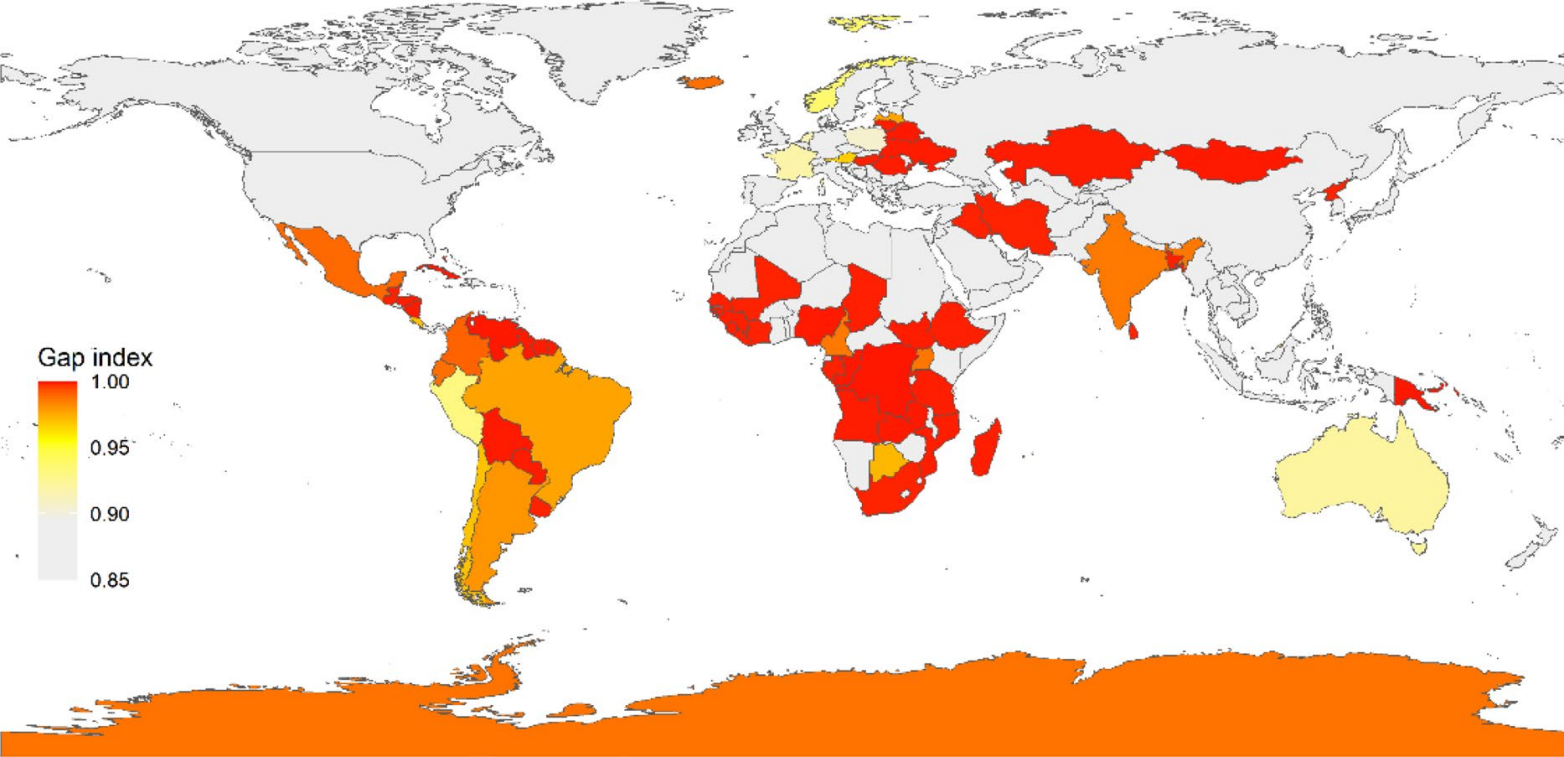
The gap index for peatland GHG flux observations in different countries. A higher index suggests a larger gap (i.e., a greater need) for observations

For CO_2_ and CH_4_, the majority of flux studies were conducted in Canada, the USA, China and Finland (> 50 studies per country) (Fig. 2a, b), followed by the UK, Russia, Sweden and Germany (> 20 studies per country). Other countries, such as Indonesia, Malaysia, Estonia, Ireland, Poland, Denmark, Peru, Panama and Japan, had more than five studies. Most of these countries have an extensive peatland cover (> 10,000 km^2^). Among the countries with limited studies (≤ 5), many have large areas of peatland, including Colombia, Argentina, Mexico, Chile, Ecuador, Norway, Latvia, Netherlands, Iceland, Uganda, Botswana, India and Australia. Many areas with substantial peatland cover had no studies at all, including many Central and South American (Honduras, Nicaragua, Cuba, Venezuela, Guyana, Suriname and French Guiana) and African countries (Senegal, Guinea-Bissau, Guinea, Sierra Leone, Nigeria, Democratic Republic of the Congo, Republic of the Congo, Zambia, Angola and Madagascar), and a few European (Lithuania, Belarus, Ukraine, Romania and Hungary) and Asian countries (Mongolia, Kazakhstan, Sri Lanka and Bangladesh) (Fig. 3).

For N_2_O, the maximum number of studies per country was 37 (China) (Fig. 2c). The majority (86%) of the studies were carried out in Europe (Finland, Germany, Sweden, Estonia and the UK), North America (the USA and Canada), Russia, China and tropical Asia (Malaysia and Indonesia). In the vast peatland areas in Central/South America, Africa and Tasmania Australia, N_2_O fluxes remain largely unstudied (Fig. 3).

### Land use type

**Fig. 4:**
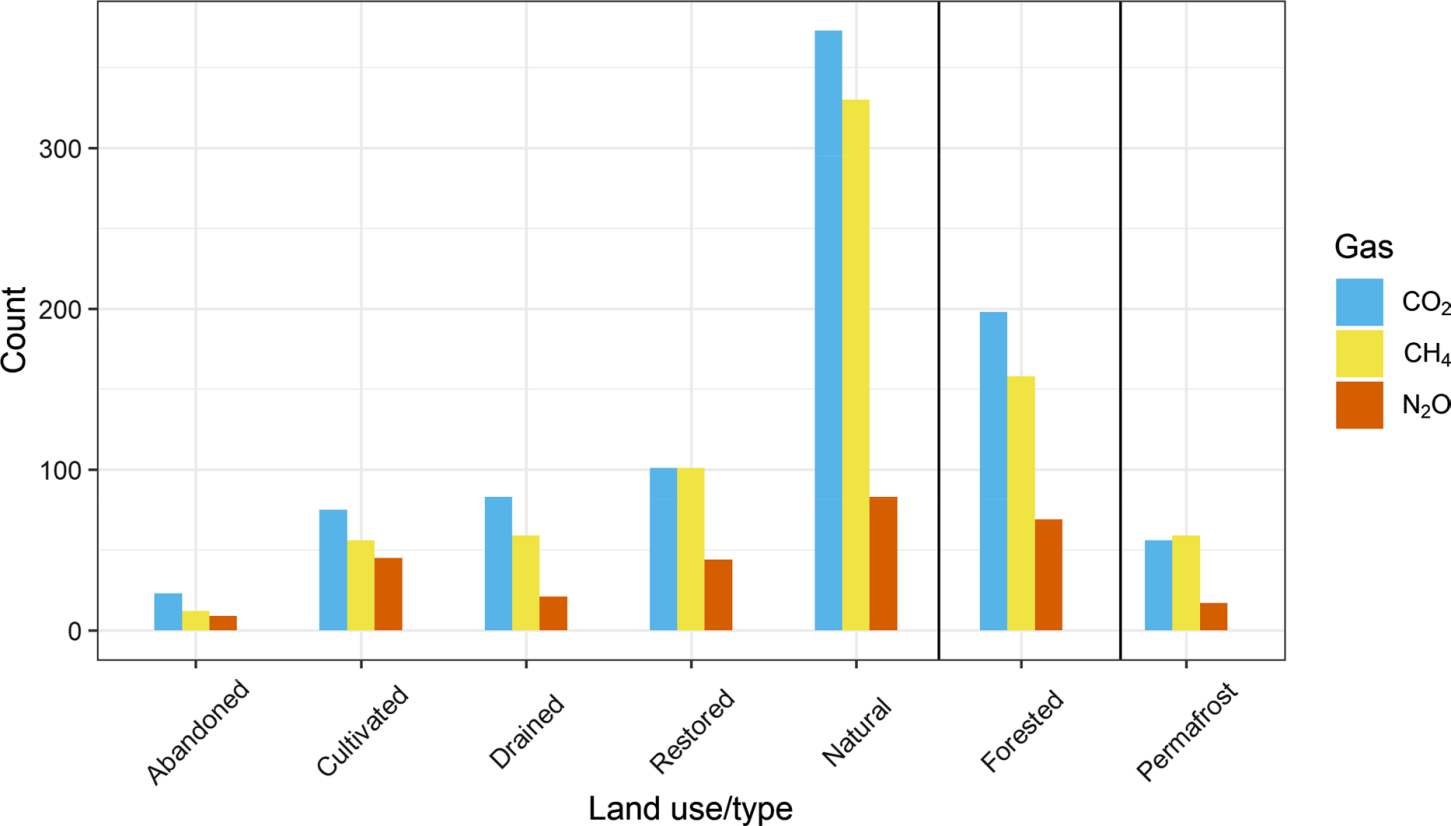
Number of peatland GHG flux studies according to land use type. Colors indicate different gas types

The majority of the studies (> 55%) that measured CO_2_ and CH_4_ fluxes were conducted in natural peatlands (Fig. 4). Studies in disturbed peatlands were carried out mainly in restored sites (~ 40%), followed by drained sites (without any management activity) (~ 27%), and cultivated sites (with crops and grasses) (~ 26%), and with minimal coverage in abandoned sites (only ~ 7%). Regardless of the land use, ~ 29% of the CO_2_ and CH_4_ flux studies were conducted in forested peatland sites and ~ 10% in permafrost sites.

Unlike for CO_2_ and CH_4_, the majority of N_2_O flux studies (59%) were conducted in disturbed/managed peatlands (Fig. 4). Among them, 18% were carried out in drained sites, 38% in cultivated sites, 37% in restored sites and only 7% in abandoned sites. Of all the N_2_O flux studies, 34% were on forested sites and 8% were on sites with permafrost.

**Fig. 5:**
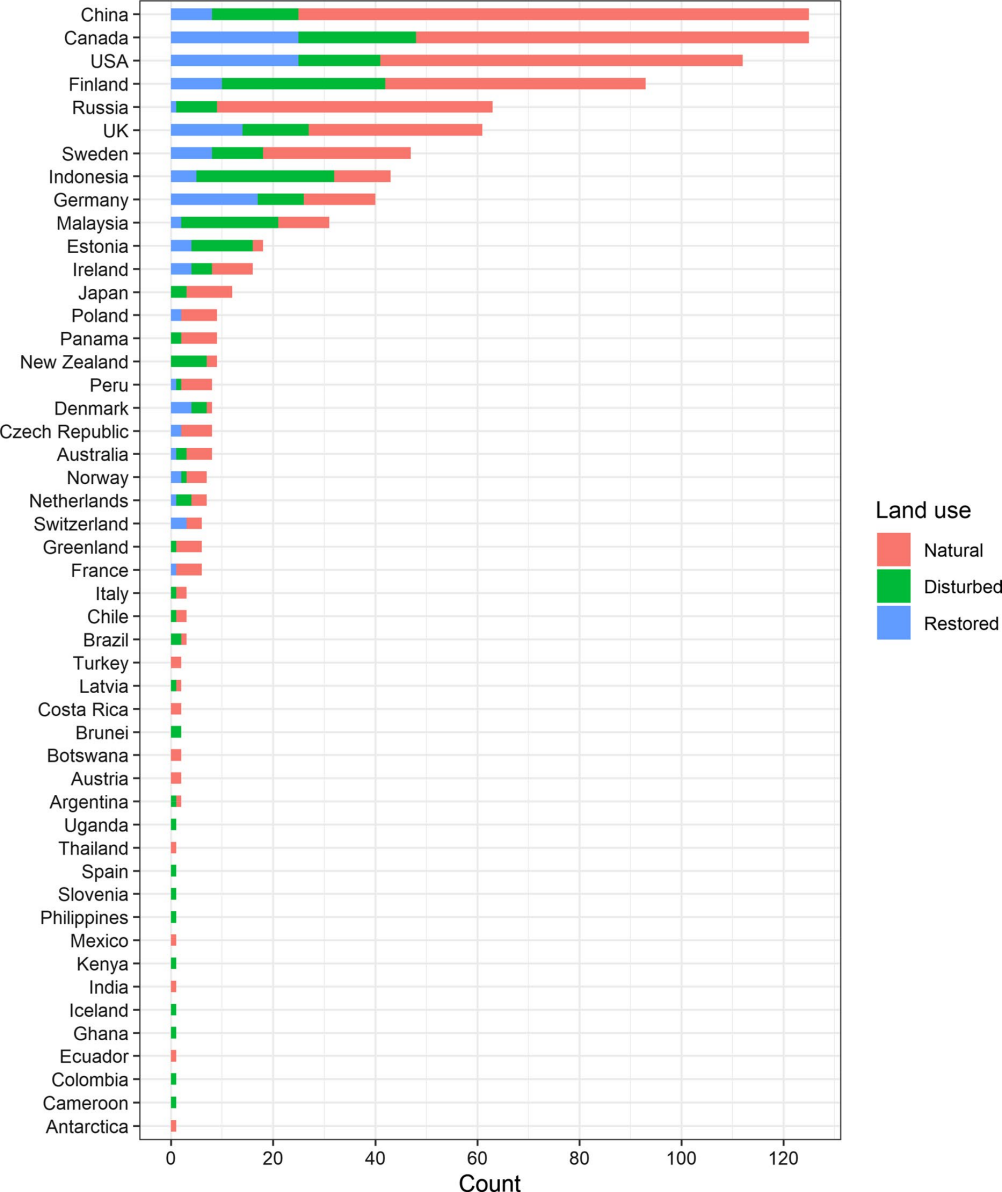
Number of peatland GHG flux studies according to country/region. Colors indicate different land use types

Among countries/regions with > 10 studies, the majority had a relatively balanced distribution across natural, disturbed, and restored ecosystems (Fig. 5). However, it should be noted that for restored ecosystems, Russia had only 1 study and Japan had none. For countries/regions with < 10 studies, there was a significant imbalance in the number of studies conducted across natural, disturbed, and restored ecosystems, where many countries/regions have exclusively studied either natural or/and disturbed ecosystems and only a few studies focused on restored ecosystems.

### Data from chamber measurements

**Fig. 6:**
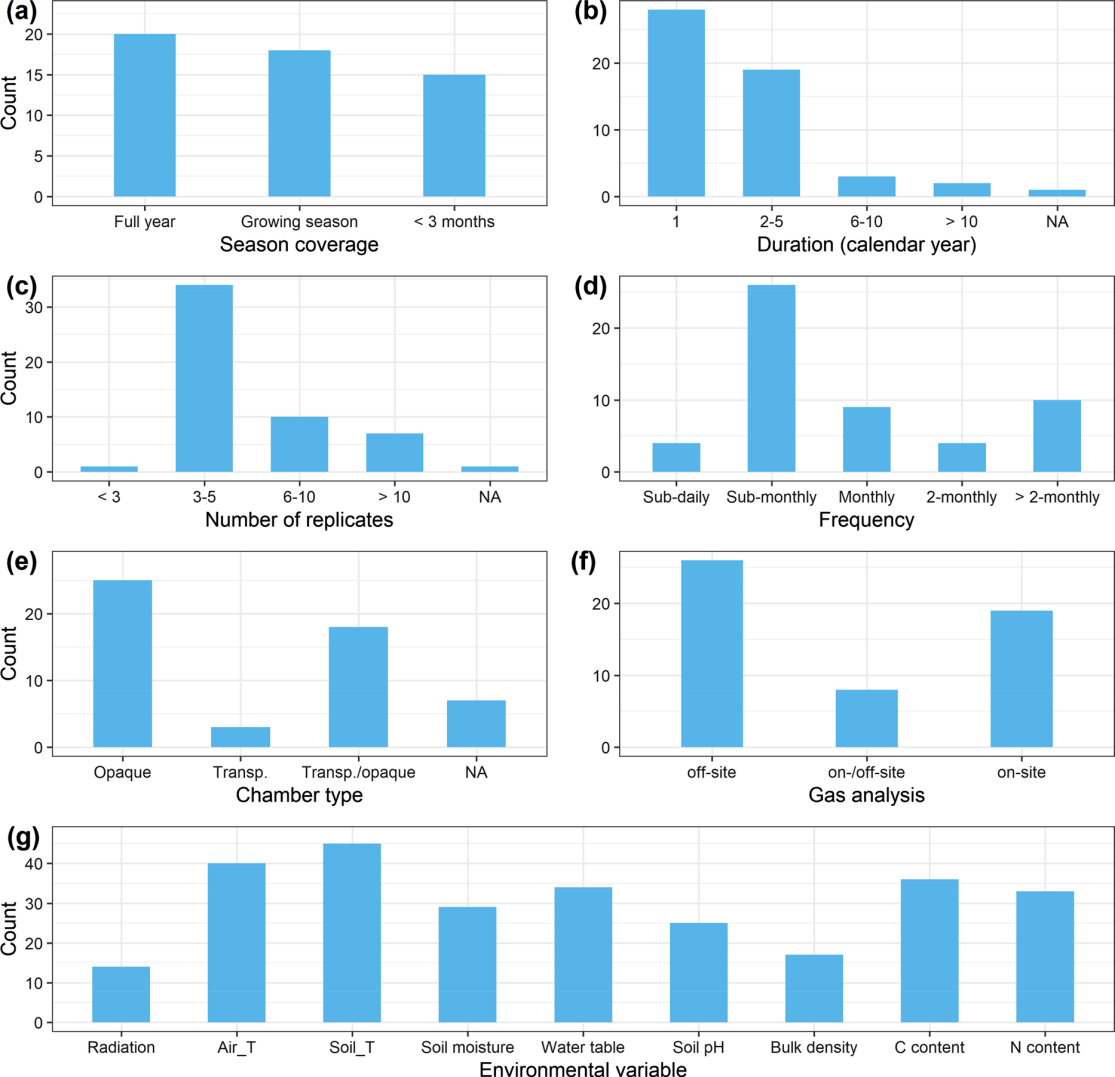
Chamber GHG flux measurements for studies published in 2022 presented by: season coverage (**a**), duration (**b**), replicates (**c**), frequency (**d**), chamber type (**e**), gas analysis (**f**) and other measured environmental variables (**g**). NA indicates that information is not reported in the paper. Air_T, air temperature. Soil_T, soil temperature. C content, carbon content. N content, nitrogen content

In our dataset, there were 53 publications in 2022 that used chambers for peatland GHG flux measurements. Among them, 20 studies (38%) covered the full year, 18 studies (34%) covered only the growing season, and 15 studies (28%) covered only periods shorter than 3 months per year (Fig. 6a). Most of the observations lasted for only 1 year or less (28 studies, 53%), 19 studies (36%) lasted for 2–5 years, only 5 studies lasted for more than 6 years and 1 study failed to document the duration (Fig. 6b). The majority of the studies (96%) had more than 3 replicates and only one study had less than 3 replicates (Fig. 6c). In terms of measurement frequency, 4 studies (8%) used automated chambers with sub-daily observations while most studies (66%) were carried out on a sub-monthly or monthly basis (Fig. 6d). There were 4 (8%) and 10 studies (19%) that measured at a frequency of 2 months and more than 2 months, respectively, including all the tropical/subtropical studies (6). Regarding the chamber types, opaque chambers were used in most studies (81%) and 40% used transparent chambers (Fig. 6e). There were also 7 studies (13%) that did not document the chamber type in the papers. Most studies (26, 49%) took gas samples and analyzed them off-site by chromatography, 19 studies (36%) measured gas concentrations continuously using on-site analyzers while 8 studies (15%) combined an on-site approach for CO_2_ flux and an off-site approach for CH_4_ and/or N_2_O fluxes (Fig. 6f). Among the key auxiliary environmental variables, air/soil temperature and soil moisture/water table were monitored in most studies (Fig. 6g); however, there were 5 (9%) and 7 studies (13%) that did not have temperature and water condition measurements, respectively. Radiation was only measured in 14 studies and, importantly, among the 21 studies (40%) using transparent chambers, 6 (11%) of them did not measure radiation. Soil pH and bulk density were only reported in 25 (47%) and 17 studies (32%), respectively. Carbon and nitrogen contents were documented in 36 (68%) and 33 studies (62%), respectively.

## Discussion

### Current status and obstacles of peatland GHG observations

Given the growing attention to the climate impact of peatlands (Frolking et al. 2011; Humpenoder et al. 2020; Loisel et al. 2021), there has been a substantial rise in the number of GHG flux observations from peatlands worldwide over the last two decades. However, our study suggests that these research efforts have been heavily concentrated in a small number of countries, whereas many regions critically lack research, including Central and South America, Africa and some Asian and European countries, despite their significant peatland coverage. There are several possible explanations for this research imbalance. The majority of observation gaps exist in developing countries, where funding for basic research and infrastructure is often insufficient. Investing resources from well-funded programs into joint research efforts and partnerships could reduce budget constraints in these areas. Nevertheless, the global value of peatland ecosystem services is the primary driver for incentivizing monitoring and research. Therefore, developing global economic incentives that value the ecosystem services provided by peatlands could encourage more sustainable and effective monitoring of these important ecosystems. In addition to economic reasons, national research policies (e.g., a low budget for GHG monitoring or peatland-relevant research) also plays a role, resulting in limited peatland studies in some economically developed countries, e.g., Norway, Netherlands and Australia.

Simultaneous measurements of CO_2_, CH_4_ and N_2_O fluxes are crucial for precisely quantifying the net GHG balance in peatlands and improving global GHG accounting (Deshmukh et al. 2023). However, we found that only 12% of the studies measured fluxes of all the three gases. Compared to CO_2_ and CH_4_, N_2_O fluxes are much less studied. N_2_O has a global warming potential 265 times greater than CO_2_ over a 100-year time horizon (IPCC 2021). Many management practices on peatlands, e.g., drainage, cultivation, nitrogen fertilization, are usually associated with high N_2_O emissions (e.g., Chaddy et al. 2019; Oktarita et al. 2017; Parn et al. 2018; Prananto et al. 2020), making peatlands important sources of N_2_O. The number of studies on N_2_O fluxes have increased significantly since 2000, but this number is still less than half of those that measured CO_2_ or CH_4_ fluxes during 2020–2022. While further research exploring N_2_O emissions from managed peatlands is crucial, it is also important to conduct more N_2_O studies on natural peatlands. Many natural peatlands usually act as a small source of N_2_O (Hergoualc’h et al. 2020; Lin et al. 2022) but their N_2_O budgets are highly uncertain due to the variability of peatland conditions and the limited data. A recent study also suggested that permafrost peatlands can be a substantial source of N_2_O, with greater emissions expected following climate-induced thawing (Voigt et al. 2020). This further highlights the importance of quantifying N_2_O emissions and understanding the feedback between the climate and peatlands.

Both EC and closed chambers are the most recognized methods for gas flux measurements (Schrier-Uijl et al. 2010; Shi et al. 2022). However, EC was used in much fewer studies compared to chamber measurements, because of its high complexity, greater equipment costs, and long-term commitment. EC is a method based on micrometeorological theories and thus has specific criteria for site selection, instrumentation and data quality control (Baldocchi 2003; Papale et al. 2006; Sabbatini et al. 2018). EC observations usually produce quality data with high temporal resolution (e.g., 30 min) and large spatial footprint (e.g., > 100 m radius from the EC tower) that are ideal for long-term ecosystem GHG monitoring and budget accounting. The high frequency measurements that integrate fluxes over a whole ecosystem also reduce the risk of missing emission “hot spots” or “hot moments”, which can be substantial in annual fluxes (Anthony et al. 2023). EC measurements are particularly valuable for forested peatlands, especially in the tropical regions, as they capture the contribution from trees; however, more studies are still needed to improve our understanding of the contributions from different components of trees (canopy and stem) using chambers (Mander et al. 2022). Moreover, although EC systems for measuring CO_2_ fluxes are sophisticated, more complete GHG budgets require additional analyzers for both CH_4_ and N_2_O, that are more expensive and add more practical constraints (e.g., Irvin et al. 2021; Staudhammer et al. 2022). These drawbacks can be compensated by future improvements in gas analyzer technology and data processing methodology. The application of the EC method has benefitted from the development of flux networks such as FLUXNET and ICOS where measurements and data processing have been standardized and data made freely available. The networks have greatly facilitated ecosystem modeling and studies of complex interactions and feedback between peatlands and climate (Helbig et al. 2022). However, since the EC observations in global peatlands are relatively limited in occurrence, future projects that expand these observations to encompass additional regions and peatland types will be highly beneficial.

Compared to EC, field campaigns utilizing chambers are a more feasible method for GHG flux measurements in remote areas where a reliable power supply is not available. However, the experimental designs employed for chamber measurements exhibit considerable variability across studies which can lead to large discrepancies in data quality (Grace et al. 2020). Opaque chambers are often used for quantification of soil respiration, N_2_O and CH_4_ fluxes due to their relative simplicity and low cost. However, investigating processes underlying CO_2_ exchange, e.g., partitioning of the heterotrophic and autotrophic respiration, requires using transparent chambers to expose vegetation to natural light and account for the contribution of photosynthesis. Opaque chambers provide only ecosystem respiration which can make comparisons of sites with different productivity difficult. Many chamber measurements rely on offsite quantification using gas chromatography, which is a more commonly available technique compared with fast gas analyzers that can quantify GHG concentration onsite. This method normally provides only a few data points (i.e., < 5) for each flux calculation, which could introduce uncertainties to the calculated fluxes. To obtain a noticeable change in gas concentrations, this method often requires a longer chamber closure time than with a fast gas analyzer, which could change the conditions within the chambers (e.g., warming) and result in biases. Potential errors due to sampling and gas sample storage also add more complications to the method (Maier et al. 2022). Thus, onsite gas analyzers are recommended for chamber measurements whenever feasible and financially viable. A significant challenge using the manual chamber technique is capturing temporal variability. Among the collected publications in 2022, many observations were conducted for less than three months per year or less frequently than once a month, especially those in the tropics. This low temporal coverage risks missing key seasonal or sub-daily events (i.e., hot moments) that can have a significant impact on the total annual budget (Anthony and Silver 2021; Regina et al. 2004). It is noted that the scarcity of tropical studies in 2022 may introduce bias to our data assessment for tropical regions. While the chamber method is helpful to investigate mechanisms underlying GHG flux at a relatively fine scale within complex ecosystems, it can also miss spatial dynamics of fluxes due to limited number of chambers that can realistically be deployed during each campaign (Dinsmore et al. 2009; Marushchak et al. 2011). In recent years, various designs of automated chambers have been used for flux measurements (e.g., Courtois et al. 2019; Mander et al. 2022). Automated chambers generate data with sub-daily temporal resolution similar to EC measurements and are more probable to capture fluxes during “hot moments”. Despite these advantages, automated chambers also have limited spatial footprint and share the disadvantages of EC in that they can be costly and require a stable power supply. Therefore, they are not likely to replace the conventional manual chamber campaigns in the short-term.

The publications collected covered natural and disturbed lands with restored peatlands being less studied in many countries (Fig. 5). In response to the climate impacts of past peatland use, several countries have implemented measures aimed at restoring disturbed peatlands (Aitova et al. 2023; Andersen et al. 2017; Chimner et al. 2017; Gonzalez and Rochefort 2019; Gunther et al. 2020). Nonetheless, the scarcity of restored peatland studies also reflects the limited restoration efforts and lack of relevant polices in many countries. The effectiveness of different approaches for restoring peatlands may differ substantially depending on factors such as peatland type, conditions, climate and use history (Darusman et al. 2023; Dohong et al. 2018; Gonzalez and Rochefort 2019; Worrall et al. 2022). Therefore, it is essential to study GHG fluxes from restored peatlands to guide and optimize future restoration approaches and plans. More GHG flux observations are particularly needed in countries with a long history of peatland use.

### Implication for modeling and global/regional upscaling

Process-based models are important tools for estimating GHG balances at regional and global scales (e.g., Bona et al. 2020; Qiu et al. 2022). To obtain accurate estimations from these models, they need to be calibrated and validated against high quality data from field observations that cover a wide range of peatlands of various types, locations, and environmental conditions. Models need high temporal resolution of GHG observations from peatlands, as well as accompanying meteorological and hydrological forcings (e.g., Fig. 6g). These high-quality data are also essential for upscaling GHG budgets to the regional and global scale via remote sensing techniques (Ingle et al. 2023). Remote sensing cannot directly measure GHG fluxes from specific ecosystems but relies on models to aid estimation of gas fluxes (Lees et al. 2018). These models are informed and validated by in-situ data. However, many areas are underrepresented by in-situ data, particularly in the low and high latitudes. The Arctic-Boreal region is “notoriously underrepresented” in the FLUXNET network (Mavrovic et al. 2023). Our study reveals a series of existing observation gaps for global peatland areas, and they could be important sources of uncertainties for modeling predictions of global GHG balance and future trajectories.

Despite great efforts made in field observations, data collected in many studies may not be suitable or sufficient for model validation. For example, flux data with inadequate temporal coverage may misrepresent the seasonal dynamics and result in biased modeling outcomes. Environmental drivers are essential for modeling the fluxes but many of them are not measured or reported in publications (Fig. 6g). Therefore, there is a need for general criteria for field GHG flux observations, particularly using the chamber method, to enhance the usability of future field data for modeling purposes. Here, we suggest a few criteria for future studies:

1. Measurements should be conducted at least monthly (weekly is recommended)
2. Last for at least one year
3. Use a minimum of three replicates
4. Report important environmental variables (see Fig. 6g)
5. Make data and adequate metadata available to users. Besides these criteria, detailed guidelines that standardize the chamber method are also needed (e.g., Charteris et al. (2020); Clough et al. (2020).

In addition, it is important for models to include a complete picture of peatland carbon/nitrogen balances to yield more accurate flux estimate. Therefore, in intensively managed systems (e.g., grassland or cropping land), large transfers of carbon or nitrogen in non-gas form (e.g., dissolved carbon in ditch water flows, organic fertilizers, lime, harvested/grazed biomass and inputs of animal excreta) and emissions associated with livestock grazing (e.g., ruminant CH_4_ emissions) should also be quantified (Campbell et al. 2021; Tiemeyer et al. 2016). Furthermore, ditches within drained peatlands have been found to exhibit highly variable GHG emissions (Minkkinen and Laine 2006) and these fluxes are seldom considered in peatland GHG balance because the spatial extent of drains has not been quantified at many sites (but see Connolly and Holden 2017; Robb et al. 2023). Drains and ditches also introduce spatial heterogeneities in water table across a peatland system, which need to be evaluated and accounted for in GHG budget estimations.

Besides GHG observations, the measurement of peat subsidence can reflect the rate of carbon loss from peatlands. The rate has been found to be closely linked to the water table drop resulting from human activities (e.g., drainage) (Hoyt et al. 2020; Ma et al. 2022). Long-term peat subsidence observations, in combination with GHG flux measurements, can provide valuable insights into the overall carbon dynamics of peatlands and is crucial for assessing the impact of peatland degradation.

### Future observation needs

Based on our review, we have summarized the key observation gaps in peatland GHG flux research that need to be addressed in future studies:

1. More GHG observations are needed in African, Central and South American countries.
2. Simultaneous measurements of fluxes of CO_2_, CH_4_, and N_2_O fluxes are essential for precisely quantifying the net GHG balance in peatlands. N_2_O studies are still too few compared to CO_2_ and CH_4_ studies, especially in natural peatland ecosystems.
3. More EC observations need to be established to achieve larger coverage and better representativeness of global peatlands for long-term monitoring.
4. The canopy role in GHG budget of peatland forests is almost unknown, therefore EC observations have a crucial role in filling this gap. It is especially important in tropical regions which are global hotspots of CH_4_ and N_2_O emissions and where most natural peatlands are swamp forests.
5. Although automated chambers are recommended for plot-scale observations, traditional field campaigns with chambers remain an essential approach for many regions. It is recommended that future studies adhere to the above-mentioned criteria to ensure good quality data.
6. There is a need for more studies on GHG fluxes in restored peatlands, with a focus on identifying optimal restoration approaches for ecosystems with different types, conditions, climates, and land use histories.

## Data Availability

The dataset of this study is available on Zenodo (10.5281/zenodo.8424351).
